# Mid-range arc therapy for efficient and RBE-robust proton treatment

**DOI:** 10.1088/1361-6560/ae674c

**Published:** 2026-05-14

**Authors:** Qingying Wang, Mingli Chen, Yinheng Zhu, Xingyi Zhao, You Zhang, Xuejun Gu, Weiguo Lu

**Affiliations:** 1 Department of Radiation Oncology, The University of Texas Southwestern Medical Center, Dallas, TX 75390, United States of America; 2 Department of Radiation Oncology and Molecular Radiation Sciences, Johns Hopkins University School of Medicine, Baltimore, MD 21231, United States of America; 3 Department of Radiation Oncology, Stanford University, Stanford, CA 94305, United States of America

**Keywords:** proton arc therapy, energy layer selection, RBE-robust, LET_d_, delivery efficiency

## Abstract

*Objective.* Proton arc therapy (PAT) has the potential to improve plan conformity while enhancing normal tissue sparing; however, its clinical translation faces practical barriers related to radiobiological uncertainties and delivery inefficiency. This study introduces a novel planning approach, mid-range PAT (MRPAT), designed to enhance delivery efficiency and mitigate range-related uncertainties in PAT. *Approach.* MRPAT employs a single mid-range energy layer per beam direction, positioning the Bragg peak near the target center (mid-range) plane to confine range uncertainty within the target while reducing dose-averaged linear energy transfer (LET_d_) and relative biological effectiveness (RBE) hotspots in adjacent organs at risk (OARs). The feasibility of MRPAT was investigated on an ellipse phantom and three clinical cases (prostate, spine, and head and neck). Physical dose distributions, LET_d_ distributions, RBE-weighted dose distributions, and delivery efficiency were compared with full-range arc employing all possible energy layers (ELs) (full-arc) and intensity-modulated proton therapy (IMPT) plans with two beams. *Main results.* Compared with IMPT, arc plans provided substantial improvements in entrance dose control and OAR sparing. The MRPAT plan achieved comparable target coverage and OAR sparing compared to the baseline full-arc plan, demonstrating the redundancy of utilizing all possible ELs. MRPAT effectively confined LET_d_ and RBE hotspots to the center of the target, reducing potential biological weighted dose spillage over the surroundings. In terms of delivery efficiency, MRPAT eliminates EL switching within the same control point by using less than 5% of the ELs, requiring only about 20% of the total beam delivery time compared with the full-arc plan. *Significance.* MRPAT demonstrates a simple, efficient, and high‐LET_d_/RBE hotspot‐containment planning concept that is practical for clinical use and adaptable to future adaptive PAT workflows.

## 1. Introduction

Proton arc therapy (PAT), delivering radiation from dozens to hundreds of angles via a rotating gantry, has been proposed as a potential advancement of proton therapy (Sandison *et al* 1997, Ding *et al* 2016, Carabe-Fernandez *et al* 2020). Compared to the conventional intensity-modulated proton therapy (IMPT) with a few static beams, PAT increases the degrees of freedom of beam angle for plan optimization, potentially allowing for further improvement of plan quality and reductions in the risk of toxicity (Seco *et al* 2013, De Jong *et al* 2023). With the advent of the pencil beam scanning (PBS) technique, which enables precise dose delivery through a spot-by-spot and layer-by-layer pattern, PBS-PAT allows the creation of highly conformal treatment plans tailored to the target (Ding *et al* 2016, Mein *et al* 2024).

Although the finite penetration depth of proton beams theoretically spares normal tissues compared with photon therapy, randomized clinical evidence demonstrating a superior clinical benefit of proton therapy remains limited (Bush *et al* 2016, Liao *et al* 2018, Hwang *et al* 2020, Lin *et al* 2020). A commonly cited reason is that the technological development of proton therapy has historically lagged behind that of photon therapy, thereby limiting opportunities to fully realize its potential advantages (Mohan and Grosshans 2017, Liao and Mohan 2018). The dominant cause of degraded proton plan quality is unmitigated range/anatomical uncertainty: the Bragg peak depends on precise patient geometry, and without volumetric image guidance to ensure plan–delivery concordance, motion and anatomical changes compromise the dose (Pham *et al* 2022). Another factor that has undoubtedly influenced proton therapy outcomes is the use of a constant relative biological effectiveness (RBE) value of 1.1 for both tumors and organs at risk (OARs) during treatment planning. RBE reflects the increased density of DNA damage induced by protons compared with photons at an equivalent dose, owing to the higher dose-averaged linear energy transfer (LET_d_) of proton irradiation. *In vitro* and animal studies have shown that LET_d_ substantially increases at the distal end of the proton range, with a corresponding rise in the actual RBE (Grassberger and Paganetti 2011, Saager *et al* 2018). Consequently, proton range uncertainty potentially not only degrades plan quality but also may lead to unintended high LET_d_/RBE dose deposition in adjacent OARs instead of the target, resulting in unnecessary normal tissue damage, reduced therapeutic effectiveness, and an increased risk of complications.

In light of these risks, the clinical impact of proton range uncertainty warrants careful consideration, and PAT is being investigated as a potential mitigation strategy. Several studies have demonstrated that with appropriate energy layer (EL) design, arc delivery can reduce the impact of range uncertainty on the physical dose distribution (Seco *et al* 2013, Chang *et al* 2020, Liu *et al* 2021). Moreover, beyond physical dose distribution, Fager *et al* (2015) and studies on monoenergetic PAT (Bertolet and Carabe 2020, Carabe *et al* 2020) have demonstrated that increasing the number of proton beams enhances LET_d_ and RBE within the target without compromising plan conformity or dose uniformity. However, distal edge tracking (Deasy *et al* 1995, 1997) which exploits the steep dose gradient to conform the target, carries an inherent risk due to elevated LET_d_ close to the target edge. Thus, an appropriately designed PAT plan should avoid distal edge tracking, as this may result in unexpected toxicities in OARs.

Another potential benefit of PAT is that rotating beam delivery enables efficient delivery to minimize the interplay effect between proton spot scanning and respiratory motion (Chang *et al* 2020, Liu *et al* 2021). Ideally, PAT would enable dynamic beam delivery with variable energies that are synchronized with continuous gantry rotation. However, most current PAT strategies rely on a discrete arc delivery mode, in which the gantry must come to a complete stop at each predefined angle to deliver a set of stacked ELs. This step-and-shoot approach significantly increases the overall beam delivery time (BDT), as it is limited by relatively long EL switching time (ELST), particularly when switching from low to high energies (Ding *et al* 2016, Li *et al* 2019, Mein *et al* 2024). To reduce BDT, several studies have adopted ELST as a simplified surrogate model, increasing EL sparsity and sequencing most EL switches downward (Liu *et al* 2020, Gu *et al* 2020, 2022, Zhang *et al* 2023). While the theory and formula are elegant, this type of PAT treatment planning optimization problem has been proven to be NP-hard (Wase *et al* 2024), exhibiting high computational complexity.

To provide a clinically practicable PAT delivery strategy that reduces the potential risk of toxicities from range uncertainty while enabling dynamic arc delivery, we developed a monoenergetic PAT planning approach that uses a mid-range EL (MREL) at each beam angle to generate PAT plans, referred to as MRPAT. The MREL is designed to place spots near the central region of the target, creating a buffer zone to account for beam range uncertainty. Further, by eliminating the energy switch at the same control point, MRPAT increases delivery efficiency. In this study, we evaluate physical dose, LET_d_, and RBE-weighted dose distributions to highlight the advantages of MRPAT in avoiding unexpected LET_d_ and RBE elevation outside the target tumor, while maintaining clinically acceptable plan quality. Additionally, we conducted a detailed analysis comparing treatment delivery times for MRPAT, Full‐Arc (using all possible ELs for each beam), and IMPT, demonstrating improved delivery efficiency with MRPAT.

## 2. Methods and materials

### 2.1. MREL selection

**Figure 1.**
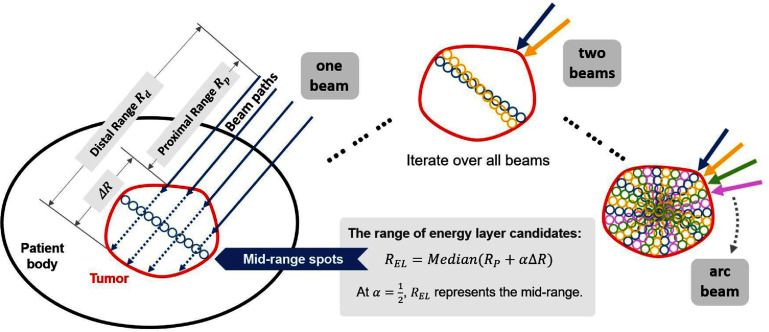
An illustrative schematic of the mid-range proton arc therapy (MRPAT) workflow. In the one beam scenario, a mid-range energy layer is delivered, represented by the small blue spots. With two beams, an additional layer of spots is introduced, improving target coverage. In the arc scenario, multiple layers are swept across the target as the gantry rotates, progressively filling the target volume with spots.

The overview workflow of MRPAT was illustrated in figure 1. MRPAT is designed to use a single EL per beam direction, positioning the Bragg peak near the central region of the target throughout the entire arc delivery. Similar to our preliminary study of MRPAT (Wang *et al* 2024), the range of MREL is selected as the median range of middle spots along each beam path by following these steps:

• Step1: Defining the potential energy deposition region. For a given beam direction, the range from source to the proximal edge ${R_{\mathrm{p}}}$ and distal edge ${R_{\mathrm{d}}}$ of the tumor can be quantified by the water equivalent path length (WEPL) obtained through patient CT scans. The potential energy deposition region along each beam path is confined between the corresponding ${R_{\mathrm{p}}}$ to ${R_{\mathrm{d}}}$, spanning a range of ${{\Delta }}R = {R_{\mathrm{d}}} - {R_{\mathrm{p}}}$.
• Step 2: determining the range of MREL. As illustrated in the one beam scenario in figure 1, the potential EL range is given by ${R_{\mathrm{p}}} + \alpha {{\Delta }}R$, where $\alpha \in \left( {0,{\text{ }}1} \right]$ is a range scaling factor. The range of MREL is determined by setting $\alpha = \frac{1}{2}$. Consequently, only one EL, MREL, is assigned to each beam direction.
• Step 3: by repeating Steps 1 and 2 for each beam direction, the MREL sequence for the arc beams is generated, as illustrated in figure 1.

At the planning stage, mid-range spots are placed on a uniform lateral grid in the beam’s-eye-view for each beam direction, consistent with standard PBS planning and our prior mid-range probing work (Chen *et al* 2018). Despite furnishing only a single EL per beam direction, the MREL has the capacity to sweep across the entire 3D grid of spots as the full arc beams provide ample spot coverage for the target, as illustrated in the arc beams scenario in figure 1. Also, as the gantry rotates, MRPAT ensures that scanning spots fill the target with Bragg peaks positioned well inside, away from the distal edge. This effectively leverages the high LET_d_ of Bragg peaks to enhance RBE in the target while reducing the risk of toxicities in the nearby OARs due to range uncertainty.

### 2.2. Phantom and clinical cases under study

The feasibility of MRPAT was first investigated using an ellipse phantom with a semi-major axis of 15 cm and a semi-minor axis of 10 cm. A *C*-shaped tumor with a radius of 3 cm was positioned at the center of the phantom, surrounding a circular OAR with a radius of 1 cm and a 0.5 cm separation from the target. This elliptical phantom geometry was designed to simulate nonuniform WEPL from the patient body surface to the target by introducing direction-dependent path lengths, in contrast to a simple cylindrical phantom with uniform thickness, thereby more closely resembling patient body contours. The phantom is water equivalent, and the densities of the target and OAR were simulated as $1.01{\text{ }}g\,\,\,{\mathrm{cm}^3}$ and $0.99{\text{ }}g\,\,\,{\mathrm{cm}^3},$ respectively. The phantom plan is prescribed 20 Gy to 95% of the planning target volume (PTV) in 10 fractions.

We further evaluated MRPAT on clinical cases, including one prostate, one spine, and one head and neck (HN) patient. The prescription guidelines for the prostate, spine, and HN cases were 70 Gy with 35 fractions, 20 Gy with 10 fractions, and 70 Gy with 35 fractions, respectively, prescribed to 95% of the PTV. All plans were optimized using physical dose-based objectives only. To ensure a fair comparison, the same set of physical dose objectives and constraints was applied across MRPAT, full-arc, and IMPT for each case. The physical dose distributions were normalized to satisfy the PTV ${D_{95\% }}$ prescription requirement. All OARs included in the optimization process for each case are listed in table S1 in the supplementary material. The optimization procedure employed quadratic objective functions to (1) achieve target coverage, (2) reduce dose to OARs using voxel-wise penalty terms, and (3) limit dose hotspots within the target.

### 2.3. RBE-weighted dose and LET_d_ calculation

A commonly used phenomenological RBE model proposed by McNamara (McNamara *et al* 2015), shown in equation (1), was used to calculated the RBE-weighted dose. The McNamara model represents a nonlinear regression fit of RBE as a function of proton physical dose ${D_{\mathrm{p}}}$ per fraction, LET_d_, and the ratio ${(\alpha /\beta )_x}$ based on the linear quadratic model. And the fitting parameters are ${p_0} = 0.99 \pm 0.01,{\text{ }}{p_1} = 0.36 \pm 0.02,{p_2} = 1.101 \pm 0.006$ and ${p_3} = - 0.0039 \pm 0.0009{\text{ }}$ (McNamara *et al* 2015).

\begin{align*}&amp; {\mathrm{RBE}}\left[ {{D_{\mathrm{P}}},{\text{ }}LE{T_{\mathrm{d}}},{\text{ }}{{\left( {\frac{\alpha }{\beta }} \right)}_x}} \right] \nonumber\\ &amp; \quad = \frac{1}{{2{D_{\mathrm{P}}}}}\left( \sqrt {\left( {\frac{\alpha }{\beta }} \right)_x^2 + 4{D_{\mathrm{P}}}{{\left( {\frac{\alpha }{\beta }} \right)}_x}\left( {{p_0} + \frac{{{p_1}}}{{{{\left( {\frac{\alpha }{\beta }} \right)}_x}}}{\mathrm{LE}}{{\mathrm{T}}_{\mathrm{d}}}} \right) + 4D_{\mathrm{P}}^2{{\left( {{p_2} + {p_3}\sqrt {{{\left( {\frac{\alpha }{\beta }} \right)}_x}} {\mathrm{LE}}{{\mathrm{T}}_{\mathrm{d}}}} \right)}^2}}\right.\nonumber\\ &amp; \qquad \left. - {{\left( {\frac{\alpha }{\beta }} \right)}_x} \right){\text{ }}.\end{align*}

The proton dose optimization and calculation were performed using our in-house planning toolkits (Lu 2010, Lu and Chen 2010, Chen *et al* 2022), which enables very large scale optimization without the need for pre-calculated beamlets. The spot spacing was 3 mm, and the control points for individual beam were spaced 5.07° resulting in totally 71 control points. LET_d_ has been considered one of the key factors for characterizing variable RBE in proton therapy (Carabe *et al* 2012, Deng *et al* 2021). In this study, LET_d_ simulations were performed using our in-house GPU-based Monte Carlo (MC) toolkit, gPMC (Jia *et al* 2011, 2012), which was validated against TOPAS (TOolkit for PArticle Simulation) version 3.9 (Perl *et al* 2012, Faddegon *et al* 2020), serving as the gold standard. Details of the validation between gPMC and TOPAS for monoenergetic proton beam LET_d_ are provided in the supplementary material. The 3D MC-based LET_d_ simulations in gPMC were conducted using the proton beam setup information, optimized beam intensities, and optimized dose distributions of both the phantom and clinical cases. The number of primary histories was set to $1 \times {10^7}$ for both gPMC validation and 3D MC-based LET_d_ simulation to ensure that the simulation results satisfied the statistical uncertainty requirement, with the relative error of the mean value kept below $1\% .$ The ${(\alpha /\beta )_x}$ ratios were considered to be 3.0 Gy for the OARs and body region and 10.0 Gy for the target tumor.

### 2.4. Robustness analysis

A scenario-based approach was adopted to evaluate the effects of range uncertainty and setup errors (Korevaar *et al* 2019). For each treatment plan, a total of eight uncertainty scenarios were generated. Range uncertainty was simulated by shifting the Bragg peak location through uniform scaling of the stopping power ratios, corresponding to ±3% WEPL, resulting in two range-uncertainty scenarios. Setup uncertainty was modeled by rigidly shifting the phantom or patient geometry by ±3 mm in the inferior-superior, right–left, and anterior–posterior directions, yielding six setup-error scenarios. For each uncertainty scenario, the physical dose, LET_d_, and RBE-weighted dose distributions were recalculated using the same optimized spot weights as in the nominal plan.

### 2.5. Delivery efficiency estimation

A simplified delivery time estimation model, described in the supplementary material, equation (S1–S5), was adopted to evaluate delivery efficiency. The total delivery time (TBD) was defined as the sum of the spot scanning time (SST), spot switching time (SSWT), ELST, and gantry rotation time (GRT) for each control point. Furthermore, to enable a general comparison independent of a specific treatment machine, we also evaluated the total number of spots, total number of ELs, and the monitor units required to deliver treatment plans across different delivery strategies.

## 3. Results

**Figure 2.**
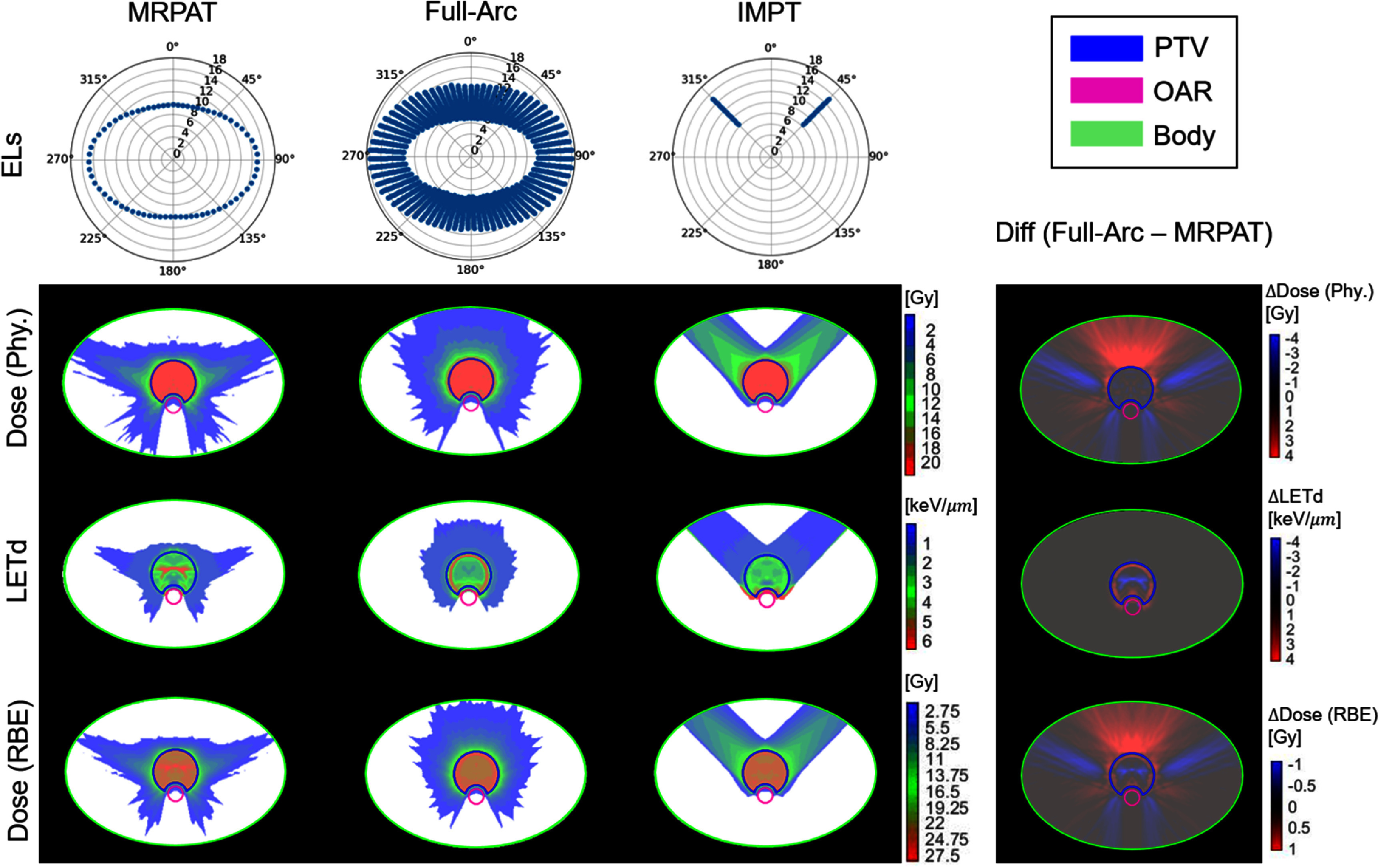
Comparison of energy layers (unit: cm), physical dose distribution, LETd and RBE-weighted dose distribution of MRPAT, full-arc, and IMPT plans for an ellipse phantom with a *C*-shaped target, with corresponding difference maps (full-arc—MRPAT).

**Figure 3.**
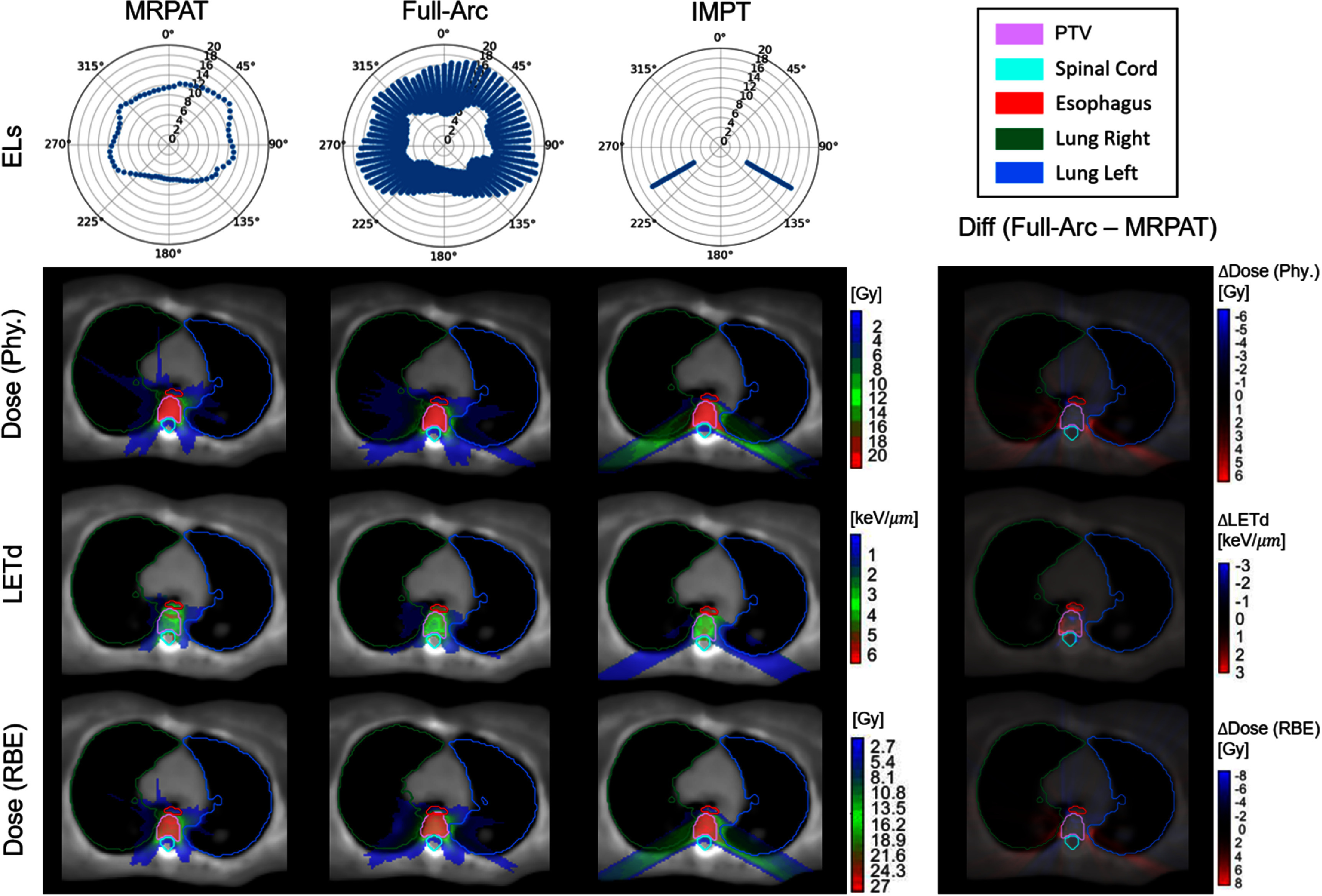
Comparison of energy layers (unit: cm), physical dose distribution, LETd and RBE-weighted dose distribution of MRPAT, full-arc, and IMPT plans for the spine case, with corresponding difference maps (full-arc—MRPAT).

**Figure 4.**
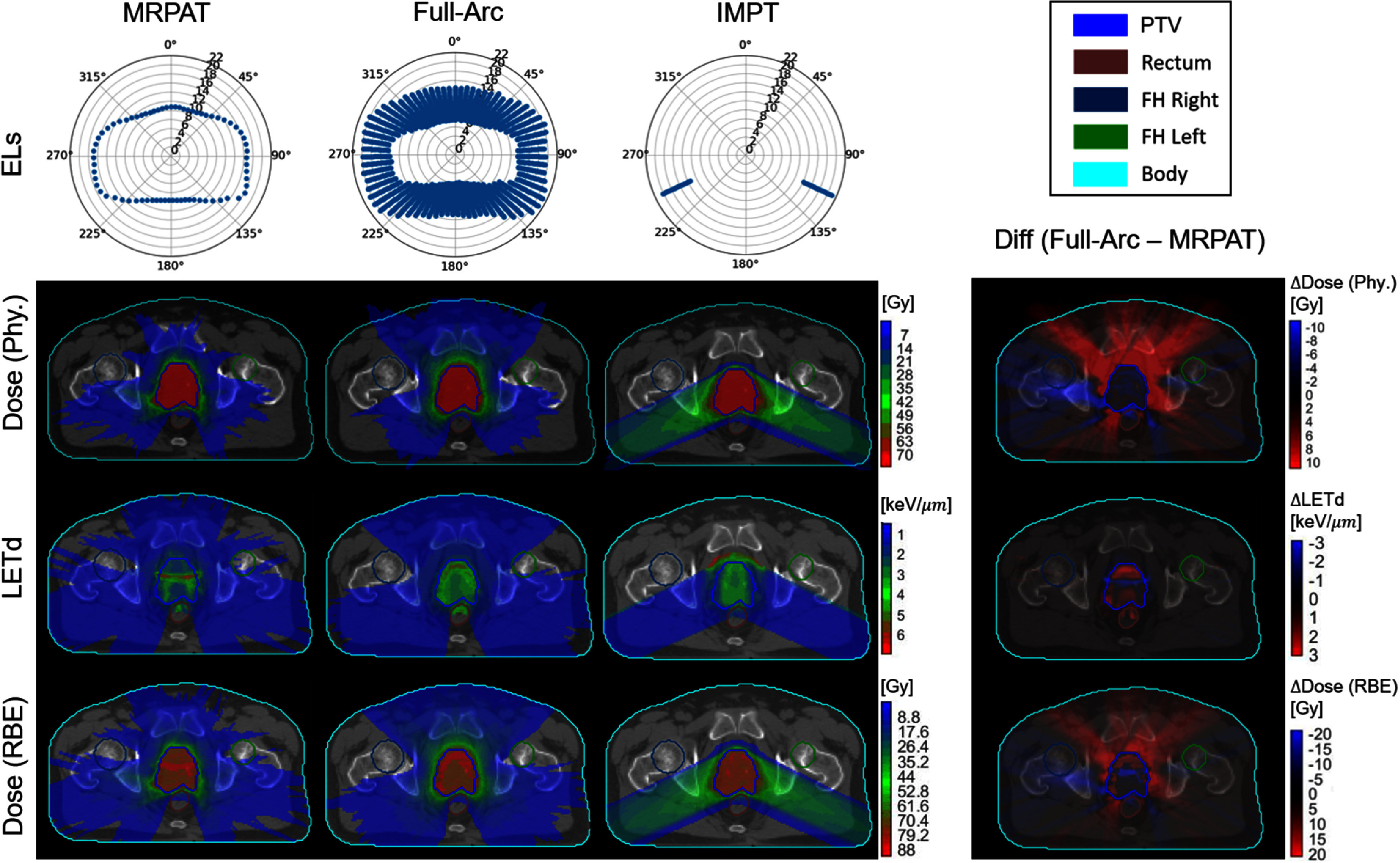
Comparison of energy layers (unit: cm), physical dose distribution, LETd and RBE-weighted dose distribution of MRPAT, full-arc, and IMPT plans for the prostate case, with corresponding difference maps (full-arc—MRPAT).

**Figure 5.**
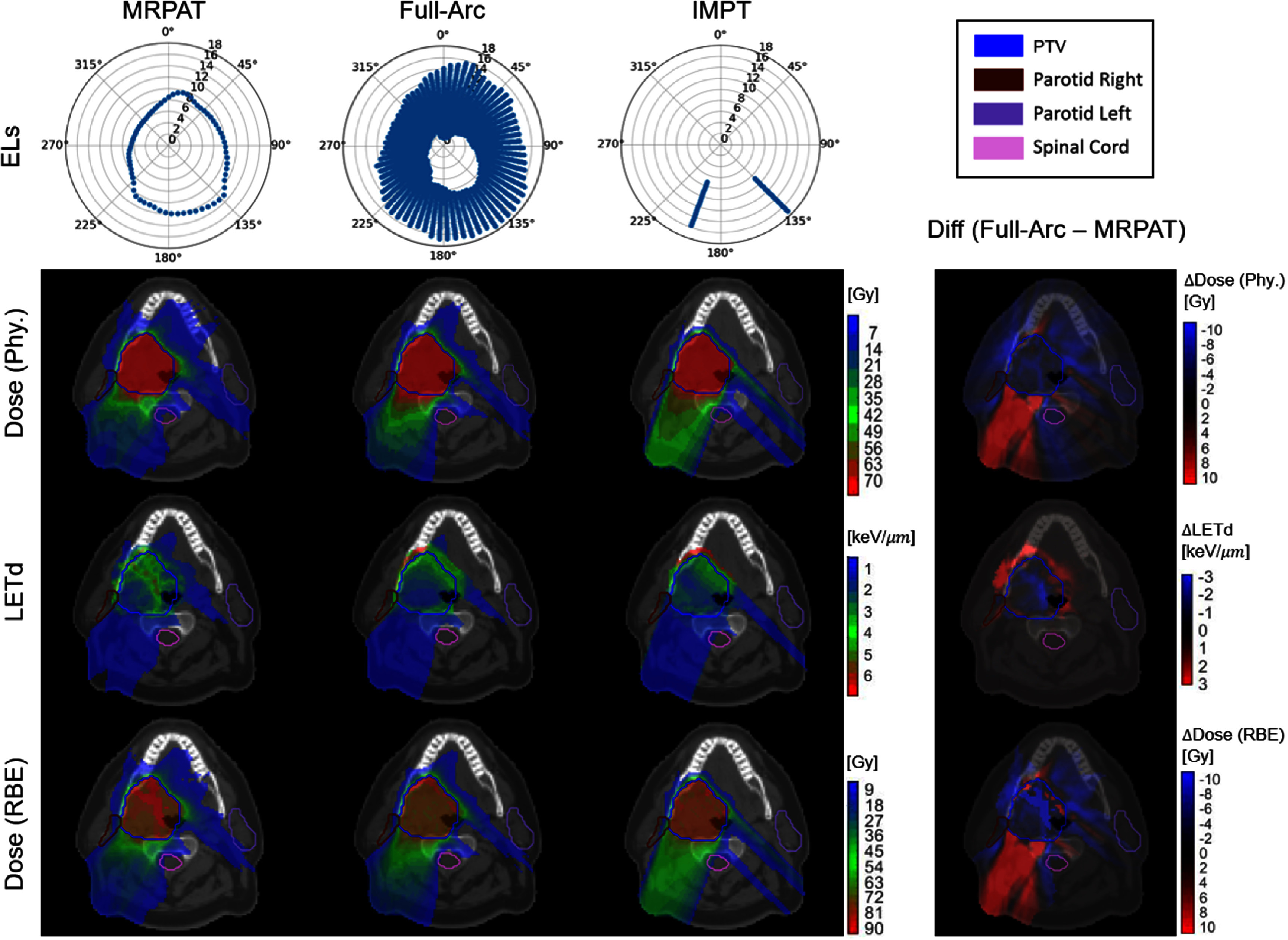
Comparison of energy layers (unit: cm), physical dose distribution, LETd and RBE-weighted dose distribution of MRPAT, full-arc, and IMPT plans for the head and neck case, with corresponding difference maps (full-arc—MRPAT).

The performance of MRPAT was compared with a baseline strategy employing full-range ELs, full-arc, and with IMPT plans utilizing two beams. Comparisons of EL arrangements, physical dose distributions, LET_d_ distributions, and RBE-weighted dose distributions for MRPAT, full-arc, and IMPT in both phantom and clinical cases are presented in figure 2–5.

**Figure 6.**
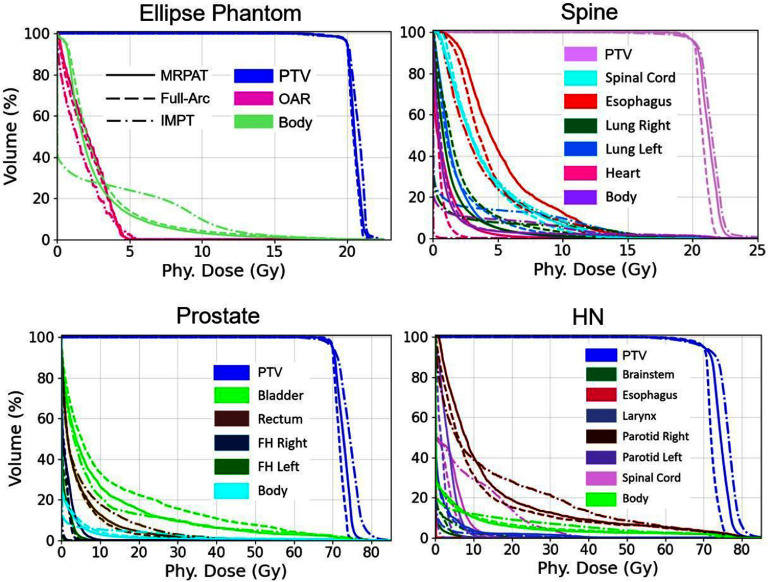
DVH comparison of MRPAT, full-arc, and IMPT plans for phantom and clinical cases.

Across phantom and clinical cases, MRPAT maintains 71 ELs for each plan and has a continuous EL pattern along the gantry rotation. In contrast, full-arc plans require more than 1000 ELs. Regarding the physical dose distributions, distinct dose patterns were observed between the arc and IMPT plans, with the IMPT plans exhibiting higher entrance doses compared with the arc plans in both phantom and clinical cases. Figure 6 shows the DVH comparisons for the MRPAT, full-arc, and IMPT plans across all cases, while corresponding DVH metrics, including ${D_{98{\% }}},{\text{ }}{D_{50{\% }}}{\text{ }}$ and ${D_{2{\% }}}$ are reported in table 1. All three planning strategies achieved comparable target coverage, whereas the arc plans provided superior OAR sparing. For instance, in the HN case, the arc plans achieved lower ${D_{2{\% }}}$ of the spinal cord compared with IMPT. The comparable plan quality between MRPAT and full-arc also suggests that spot utilization is highly redundant in arc beams with full energy.

**Table 1.** DVH metric comparisons among MRPAT, full-arc, and IMPT plans of ellipse phantom, spine, prostate, and head and neck cases. ${D_{x\% }}$ (Gy) represents the dose that *x*% of the volume of the PTV or OAR is at least receiving.

Ellipse phantom						

	MRPAT		Full-arc		IMPT	
	${D_{98\% }}$	${D_{2\% }}$	${D_{98\% }}$	${D_{2\% }}$	${D_{98\% }}$	${D_{2\% }}$
PTV	19.58	21.33	19.72	21.10	19.48	21.53
	${D_{50\% }}$	${D_{2\% }}$	${D_{50\% }}$	${D_{2\% }}$	${D_{50\% }}$	${D_{2\% }}$
OAR	2.13	4.45	1.92	4.56	1.36	5.03

Spine case						

	MRPAT		Full-arc		IMPT	

	${D_{98\% }}$	${D_{2\% }}$	${D_{98\% }}$	${D_{2\% }}$	${D_{98\% }}$	${D_{2\% }}$
PTV	19.49	22.60	19.60	21.84	19.23	23.05
	${D_{50\% }}$	${D_{2\% }}$	${D_{50\% }}$	${D_{2\% }}$	${D_{50\% }}$	${D_{2\% }}$
Esophagus	4.46	13.91	3.54	12.17	2.65	14.67
Spinal canal	2.90	12.28	3.00	11.05	2.98	12.85
Lung right	0.83	7.73	1.39	11.43	0.04	15.33
Lung left	1.17	7.32	1.05	9.71	0.04	15.11

Prostate case						

	MRPAT		Full-arc		IMPT	

	${D_{98\% }}$	${D_{2\% }}$	${D_{98\% }}$	${D_{2\% }}$	${D_{98\% }}$	${D_{2\% }}$
PTV	68.94	76.26	69.45	74.17	67.95	80.43
	${D_{50\% }}$	${D_{2\% }}$	${D_{50\% }}$	${D_{2\% }}$	${D_{50\% }}$	${D_{2\% }}$
Bladder	3.86	69.27	5.31	70.16	3.29	70.60
Rectum	1.75	28.35	1.77	23.62	1.67	34.00
Femoral head right	0.45	7.04	0.59	3.34	0.13	0.73
Femoral head left	0.12	4.84	0.20	3.97	0.13	0.38

HN case						

	MRPAT		Full-arc		IMPT	

	${D_{98\% }}$	${D_{2\% }}$	${D_{98\% }}$	${D_{2\% }}$	${D_{98\% }}$	${D_{2\% }}$
PTV	65.90	79.18	67.16	76.07	64.52	81.78
	${D_{50\% }}$	${D_{2\% }}$	${D_{50\% }}$	${D_{2\% }}$	${D_{50\% }}$	${D_{2\% }}$
Spinal cord	0.44	12.94	0.34	9.44	0.78	34.02
Brainstem	0.15	3.96	0.15	5.04	0.14	11.00
Parotid right	3.27	9.43	1.61	5.63	0.14	1.32

The most notable advantage of MRPAT is reflected in the LET_d_ and RBE-weighted dose distributions. Across all phantom and clinical cases, MRPAT plans tended to localize the high region of LET_d_ within the central portion of the target, whereas full-arc plans produced high LET_d_ near the target edge. This distinction is further demonstrated in the corresponding difference maps (full-arc − MRPAT), where positive LET_d_ differences are observed near the target boundary, indicating higher LET_d_ values in the full-arc strategy in these regions. In contrast, IMPT plans demonstrated high LET_d_ spillages extending into adjacent normal tissues, posing a greater risk of biological toxicity. The RBE-weighted dose exhibited a similar pattern to the LET_d_ distribution, with MRPAT generating a distinct RBE-weighted dose boost concentrated in the central portion of the target. To further quantify the hotspot spillage, the two percentile values of LET_d_ and RBE-weighted dose for all cases are provided in table 2. These quantitative results indicate that MRPAT plans exhibit higher hotspot values of LET_d_ and RBE-weighted dose within the PTV compared with full-arc and IMPT plans in most cases. More importantly, MRPAT achieved lower high LET_d_ in most OARs and the surrounding body regions across both phantom and clinical cases.

**Table 2.** Hotspot values of LET_d_ and RBE weighted dose (${D_{{\mathrm{RBE}}}}$) for MRPAT, full-arc, and IMPT plans, evaluated on the ellipse phantom, spine, prostate, and head and neck (HN) cases. Here we use 2 percentile value to account for MC dose uncertainty. Bold values show the highest LET_d_ and ${D_{{\mathrm{RBE}}}}$ values in the entire body.

Ellipse phantom						

	MRPAT		Full-arc		IMPT	
	${\left( {{\mathrm{LE}}{{\mathrm{T}}_{\mathrm{d}}}} \right)_{2\% }}$	${\left( {{D_{{\mathrm{RBE}}}}} \right)_{2\% }}$	${\left( {{\mathrm{LE}}{{\mathrm{T}}_{\mathrm{d}}}} \right)_{2\% }}$	${\left( {{D_{{\mathrm{RBE}}}}} \right)_{2\% }}$	${\left( {{\mathrm{LE}}{{\mathrm{T}}_{\mathrm{d}}}} \right)_{2\% }}$	${\left( {{D_{{\mathrm{RBE}}}}} \right)_{2\% }}$
PTV	**6.60**	**27.83**	**6.13**	**27.40**	**5.46**	**26.68**
OAR	0.01	5.64	0.01	5.11	0.01	5.89
Body	1.56	12.47	1.83	14.69	1.52	15.40

Spine case						

	MRPAT		Full-arc		IMPT	

	${\left( {{\mathrm{LE}}{{\mathrm{T}}_{\mathrm{d}}}} \right)_{2\% }}$	${\left( {{D_{{\mathrm{RBE}}}}} \right)_{2\% }}$	${\left( {{\mathrm{LE}}{{\mathrm{T}}_{\mathrm{d}}}} \right)_{2\% }}$	${\left( {{D_{{\mathrm{RBE}}}}} \right)_{2\% }}$	${\left( {{\mathrm{LE}}{{\mathrm{T}}_{\mathrm{d}}}} \right)_{2\% }}$	${\left( {{D_{{\mathrm{RBE}}}}} \right)_{2\% }}$
PTV	**5.35**	**28.07**	4.95	**27.34**	4.72	**28.47**
Esophagus	4.49	15.16	**5.40**	14.08	**5.27**	16.69
Spinal canal	5.06	13.65	4.08	12.26	4.18	14.11
Lung right	0.36	7.89	0.67	11.62	0.47	15.64
Lung left	0.26	7.46	0.62	9.93	0.77	15.50
Body	1.46	10.92	1.72	11.62	1.32	15.67

Prostate case						

	MRPAT		Full-arc		IMPT	

	${\left( {{\mathrm{LE}}{{\mathrm{T}}_{\mathrm{d}}}} \right)_{2\% }}$	${\left( {{D_{{\mathrm{RBE}}}}} \right)_{2\% }}$	${\left( {{\mathrm{LE}}{{\mathrm{T}}_{\mathrm{d}}}} \right)_{2\% }}$	${\left( {{D_{{\mathrm{RBE}}}}} \right)_{2\% }}$	${\left( {{\mathrm{LE}}{{\mathrm{T}}_{\mathrm{d}}}} \right)_{2\% }}$	${\left( {{D_{{\mathrm{RBE}}}}} \right)_{2\% }}$
PTV	**5.08**	**94.18**	**4.69**	**91.88**	3.77	**91.95**
Rectum	3.88	31.43	3.93	26.53	2.24	36.57
Bladder	4.95	75.90	4.22	77.98	**4.13**	76.04
Femoral head right	0.01	7.09	0.01	3.36	0.01	0.76
Femoral head left	0.01	4.84	0.01	4.02	0.01	0.15
Body	0.97	13.54	1.06	17.54	1.06	32.89

HN case						

	MRPAT		Full-arc		IMPT	

	${\left( {{\mathrm{LE}}{{\mathrm{T}}_{\mathrm{d}}}} \right)_{2\% }}$	${\left( {{D_{{\mathrm{RBE}}}}} \right)_{2\% }}$	${\left( {{\mathrm{LE}}{{\mathrm{T}}_{\mathrm{d}}}} \right)_{2\% }}$	${\left( {{D_{{\mathrm{RBE}}}}} \right)_{2\% }}$	${\left( {{\mathrm{LE}}{{\mathrm{T}}_{\mathrm{d}}}} \right)_{2\% }}$	${\left( {{D_{{\mathrm{RBE}}}}} \right)_{2\% }}$
PTV	4.71	**96.00**	4.26	**92.70**	**3.66**	**91.22**
Spinal cord	0.01	13.10	0.01	9.55	1.02	34.45
Brainstem	0.01	3.95	0.01	5.10	0.02	11.14
Parotid right	**5.32**	82.53	**4.93**	81.08	2.58	81.68
Body	2.09	71.49	2.16	74.82	1.83	72.74

**Figure 7.**
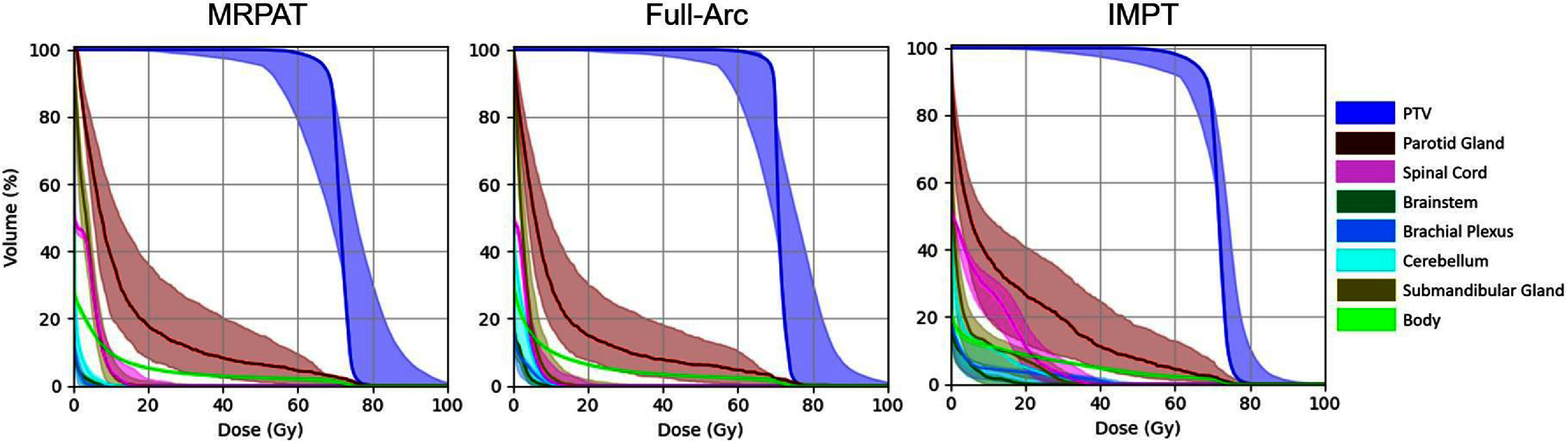
Scenario-based robustness analysis of physical dose DVHs for MRPAT, full-arc, and IMPT plans for the HN case. The nominal solution is shown as a solid line, and the shaded band represents the worst-case dose distributions across all range uncertainty and setup error scenarios.

**Figure 8.**
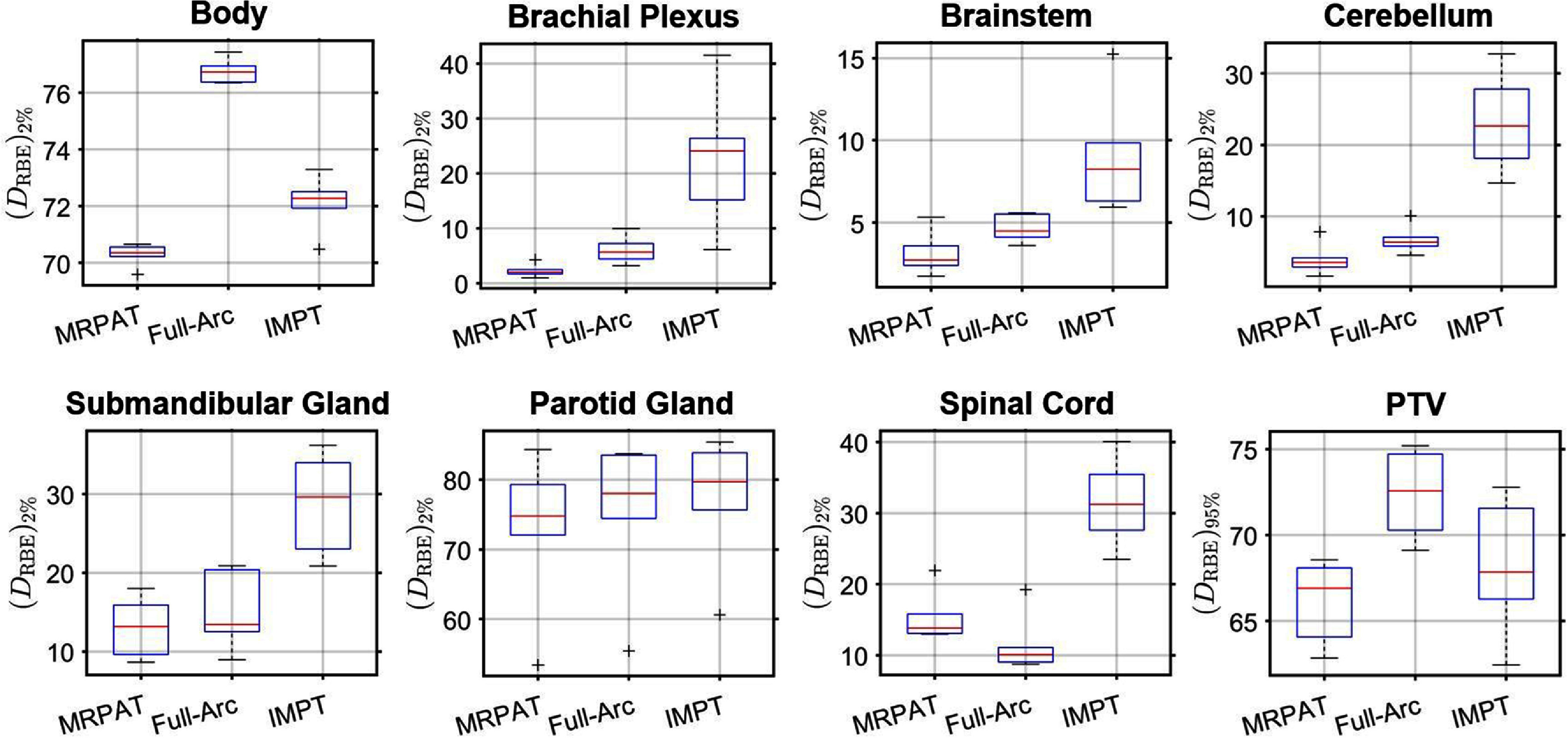
Robustness analysis comparison of target coverage (D_RBE)_(95%) and (D_RBE)_(2%) for seven OARs for the HN case. The robustness analysis was performed with a scenario-based approach over 8 scenarios. The middle line in the box plot represents the median of the distribution, the upper and lower lines represent the 5th and 95th percentile.

Figures 7 and 8 present the scenario-based robustness evaluation for the HN case. Figure 7 illustrates the DVH bands of physical dose, where the solid curves represent the nominal plans and the shaded regions delineate the worst-case DVH across all range and setup uncertainty scenarios. Similar DVH bandwidths for both arc plans, whereas IMPT exhibited broader DVH bandwidths for the parotid gland and spinal cord. Figure 8 further presents the robustness comparison based on RBE-weighted dose, which intrinsically reflects the combined effects of physical dose and LET_d_. For most OARs, MRPAT consistently exhibits lower worst-case and median ${\left( {{D_{{\mathrm{RBE}}}}} \right)_{2\% }}$ values compared with full-arc and IMPT plans. In addition, the spread of ${\left( {{D_{{\mathrm{RBE}}}}} \right)_{2\% }}$ across uncertainty scenarios is generally smaller for MRPAT, indicating reduced sensitivity to setup and range uncertainties. For the PTV, MRPAT shows slightly lower ${\left( {{D_{{\mathrm{RBE}}}}} \right)_{95\% }}$ compared with full-arc and IMPT plans.

The detailed delivery efficiency comparison for both phantom and clinical cases, including relative metrics (Total MU, ELs, and spot numbers) and machine-specific time metrics (SST, SSWT, ELST, GRT, and TBD), is presented in table 3. Under the assumption of our simplified time model, ELST is the primary contributor to the total beam delivery time. A key advantage of MRPAT is its elimination of EL switching at one control point, significantly improving delivery efficiency. Compared to full-arc plans, the MRPAT plan utilized less than 5% of the ELs and required only about 20% of the TBD time.

**Table 3.** Comparison of delivery efficiency per fraction for MRPAT, full-arc, and IMPT plans, for the ellipse phantom and three clinical cases (prostate, spine, and head and neck cases).

	Ellipse phantom			Spine case			Prostate case			HN case		
	MRPAT	Full-arc	IMPT	MRPAT	Full-arc	IMPT	MRPAT	Full-arc	IMPT	MRPAT	Full-arc	IMPT
Total MU	162.93	203.76	181.06	3311.10	4031.80	3728.70	7258.80	9690.80	8602.50	21 201	23 021	22 347
Energy layer number	71	1531	46	71	1841	68	71	1807	47	71	2280	58
Spot number	1462	16 673	742	12 580	109 662	7297	9481	153 143	3945	25 320	222 880	16 077
SST (sec)	4.05	5.09	4.53	16.55	20.16	18.64	36.20	48.45	43.01	106.05	115.10	111.73
SSWT (sec)	2.92	33.34	1.48	25.16	219.32	14.59	18.96	306.28	7.89	50.64	445.76	32.15
ELST (sec)	0.00	876.00	26.40	0.00	1062	39.60	0.00	1041.60	27.00	0.00	1325.40	33.60
GRT (sec)	283.76	390.50	11.00	289.69	390.50	11.00	289.69	390.50	11.00	277.83	390.50	11.00
TBD (sec)	290.73	1304.90	43.41	331.40	1692.00	83.84	344.85	1786.80	88.90	434.52	2276.80	188.49

## 4. Discussion

This study presents a novel MRPAT framework that employs the mono-energetic pre-selected MREL per beam direction to reduce potential elevated LET_d_ and RBE associated with range uncertainty and address challenges of delivery efficiency in PAT. The MRPAT framework employs a heuristic approach in which EL selection is performed prior to spot intensity optimization, with the goal of achieving adequate target coverage while ensuring safe and efficient delivery.

Current results from both phantom and clinical cases demonstrate that the MRPAT framework is able to generate plans with clinically acceptable dosimetric quality, high delivery efficiency with potential continuous arc delivery, and strong safety performance by effectively protecting adjacent OARs from toxicity risks while confining LET_d_ and RBE within the target. As presented in table 2, the MRPAT plan successfully confined the hotspots within the target region in most cases. An exception occurred in the parotid gland of the HN case, where the right parotid was immediately adjacent to PTV, requiring a steep dose gradient and resulted in elevated LET_d_. This effect could potentially be mitigated by incorporating LET_d_-based optimization (Deng *et al* 2021). Interestingly, as shown in figures 2–4, the MRPAT plans exhibited a distinct LET_d_ pattern in the ellipse phantom, spine, and prostate cases, characterized by a horizontal band across the central portion of the target rather than a circular hotspot region. This phenomenon primarily arises from the dependence of LET_d_ peak values on beam energy. For instance, in the ellipse phantom, to position the Bragg peak at the target center, lateral beams (from the left and right) require higher energies than anterior and posterior beams. Since the LET_d_ peak decreases with increasing energy, the anterior and posterior beams contribute more prominently to the overall LET_d_ distribution. In addition, body geometry, particularly the relatively flat posterior surface in most patients, also influences the spatial shape of the LET_d_ hot region. Notably, the scenario-based robustness evaluation demonstrated that MRPAT maintains stable target coverage while effectively limiting uncertainty-induced RBE-weighted dose spillage into adjacent OARs under range uncertainty and setup error scenarios. This robustness can be attributed to the mid-range energy selection strategy, in which even spots located near the lateral boundary of the target may have Bragg peaks positioned well inside the target along the beam direction, leaving sufficient proximal and distal buffer to accommodate range perturbations. As a result, elevated LET_d_ and RBE hotspots remain confined within the target under uncertainty, in contrast to full-arc strategy where Bragg peaks are often placed near the distal target boundary and are therefore more susceptible to range errors. For mono-energetic delivery strategies, target coverage under uncertainty can be sensitive to perturbations. However, the angular averaging inherent to arc delivery in MRPAT provides effective compensation. In practice, the use of dual or multi-arc configurations could further enhance target robustness by improving dose conformity and error compensation without substantially increasing delivery complexity (Engwall *et al* 2022). While robust optimization was not explicitly employed in this study, the MRPAT framework is inherently compatible with robust optimization techniques. The pre-selection of a MREL per beam direction establishes a well-defined energy structure that can be readily integrated with robust objective functions to further improve robustness against range and setup uncertainties. The combination of MRPAT with explicit robust optimization will be explored in future work.

Previous monoenergetic PAT studies have demonstrated dosimetric feasibility in brain tumors (Sánchez-Parcerisa *et al* 2016, Bertolet and Carabe 2020) and chordomas located at the base of the skull (Blanco Kiely and White 2016), and highlighted the inherently efficient delivery characteristics of monoenergetic PAT. Beyond that, MRPAT introduces a proof-of-concept monoenergetic strategy that emphasizes the importance of incorporating range uncertainty considerations into EL arrangement. The MREL concept is specifically developed to account for and mitigate the uncertainty of high LET_d_ or RBE spillage by terminating the beam within the target, thus confining range-related uncertainties to its interior. Moreover, a commonly monoenergetic strategy is to divide the arc beams into several sectors, within which either a single fixed EL is applied (Bertolet and Carabe 2020) or a filtered EL sequence is employed that switches from higher to lower energies within each sector (Engwall *et al* 2022, Wuyckens *et al* 2024). A potential concern with these strategies is the presence of large energy jumps between adjacent sectors, which can exacerbate range uncertainty and increase sensitivity to respiratory motion during delivery. Conversely, the MRPAT plan employs a smoothly varying MREL that follows the continuous WEPL change, effectively eliminating such EL transitions and enhancing delivery robustness.

The MRPAT also presents a simple, practical, and biologically favorable approach for PAT planning. Its simplicity lies in the direct use of a single MREL per beam direction, which substantially reduces the number of candidate spots to be optimized and eliminates the need for complex energy sequencing or multi-objective optimization (Gu *et al* 2020). This design greatly reduces planning complexity and computation time. Using our in-house planning system (Lu 2010, Lu and Chen 2010, Chen *et al* 2022), the entire MRPAT process, including MREL selection, dose optimization, and dose calculation, takes approximately 25 s per clinical case in this study. This relatively short planning time demonstrates the potential of MRPAT for integration into time-sensitive adaptive and online adaptive PAT workflows (Paganetti *et al* 2021, Draguet *et al* 2024). Moreover, the current results demonstrate that such a simplified strategy can effectively handle convex tumors and concave (*C*-shaped) targets as investigated in this study. Nevertheless, as tumor geometry becomes increasingly irregular or highly concave, maintaining uniform dose coverage and biological enhancement may become more challenging, warranting further evaluation in future studies. Additionally, the present MRPAT framework does not explicitly include LET_d_ or RBE terms in the inverse optimization, the observed biological boosting effect arises purely from the MREL arrangement and physical dose optimization. Despite this, MRPAT inherently confines high LET_d_ and RBE regions within the target, effectively avoiding hotspots spillage into adjacent normal tissues. This feature underscores compatibility of MRPAT with LET_d_ or RBE-guided optimization paradigms (Sánchez‐Parcerisa *et al* 2019, Deng *et al* 2021, McIntyre *et al* 2023), suggesting its potential as an efficient foundation for future LET_d_-painting strategies (Bassler *et al* 2010, 2014) that aim to biologically intensify the target while ensuring robust and safe delivery.

As for the delivery efficiency, the key limitation that MRPAT aims to overcome is the difficulty in synchronizing energy switching with gantry rotation. Our MRPAT strategy eliminates EL switching at one control point, ensuring that EL changes occur only during gantry rotation from the current control point to the next. Additionally, the smooth energy variation of MREL would not increase the delivery time as energy switching can take place during gantry rotation with a typically gantry rotation speed ranging from 0.1° sec^−1^ to 6° sec^−1^ (Li *et al* 2019, Liu *et al* 2020). The current delivery time estimation is based on a simplified time model with a constant gantry rotation speed and disregards any dead time in PAT delivery. While designing an accurate time model is out of the scope of this study, future improvement in efficiency through optimizing gantry rotation speeds to accommodate MREL sequence is worthwhile to be considered. Additional feasible approach for further improving delivery efficiency is adding a post-filter for MREL (Wuyckens *et al* 2024). This filter could be designed to remove low-weighted ELs that involve upward switching, achieving a balance between ELST and an acceptable sacrifice in dosimetric quality. A recent study presents another avenue to achieve faster delivery by using a tertiary energy modulator (EM) at the nozzle to externally modulate the energy of a single energy beam (Modiri *et al* 2024). The EM modulation could be guided by the MRPAT strategy, leveraging the MREL continuous nature to facilitate relatively smooth energy modulation movement.

## 5. Conclusions

This study presents MRPAT, a novel spot‐scanning arc delivery strategy that employs a single MREL per beam direction to address key challenges of delivery efficiency in PAT. By placing the Bragg peak near the center of the target, MRPAT enhances delivery robustness, confines range-related uncertainties within the target, and reduces high‐LET_d_ and RBE hotspots spillage in surrounding normal tissues. With its compatibility for integration into existing clinical workflows, MRPAT provides a proof-of-concept pathway toward an efficient, uncertainty-aware, and biologically informed PAT, paving the way for future adaptive and online treatment workflows.

## Data Availability

The data cannot be made publicly available upon publication because they contain sensitive personal information. The data that support the findings of this study are available upon reasonable request from the authors. Supplementary Information available at https://doi.org/10.1088/1361-6560/ae674c/data1.
