# Supplementary material for: Mid-Range Arc Therapy for Efficient and RBE-Robust Proton Treatment
Source: Phys Med Biol. Author manuscript; Available in PMC 2026 Jun 17. (PMC13173004; doi:10.1088/1361-6560/ae674c)
Supplement: Supplementary Material [file NIHMS2174492-supplement-Supplementary_Material.docx]

Supplementary Material

This document contains supplementary results to accompany the manuscript named: “Mid-Range Arc Therapy for Efficient and RBE-Robust Proton Treatment”.

1. Organs at Risk (OAR) Included in Treatment Planning

Table S1. OARs included in the treatment planning for the spine, prostate, and head and neck (HN) cases.

| **Spine Case** | **Prostate Case** | **HN Case** | |
| --- | --- | --- | --- |
| Lung | Femoral Head | Cavity Oral | Spinal Cord |
| Heart | Rectum | Mandible | Submandibular Gland |
| Spinal Cord | Bladder | Cerebellum | Brachial Plexus |
| Esophagus |  | Parotid Gland | Soft Palate |
|  |  | Brainstem | Esophagus |
|  |  | Larynx | Cochlea |

1. Validation of LET_d_ Monte Carlo simulation by gPMC

A 150 MeV monoenergetic proton beamlet with a $5\times5 mm^{2}$ spot size was simulated in a $25 \times25 \times25 cm^{3}$ water phantom using both gPMC and TOPAS. The number of primary histories was set to $1\times{10}^{7}$ to ensure that the simulation results satisfied the statistical uncertainty requirement, with the relative error of the mean value kept below $1\%.$ The LET_d_ scoring method follows the descirption by Granville and Sawakuchiby *(Granville and Sawakuchi 2015)*.


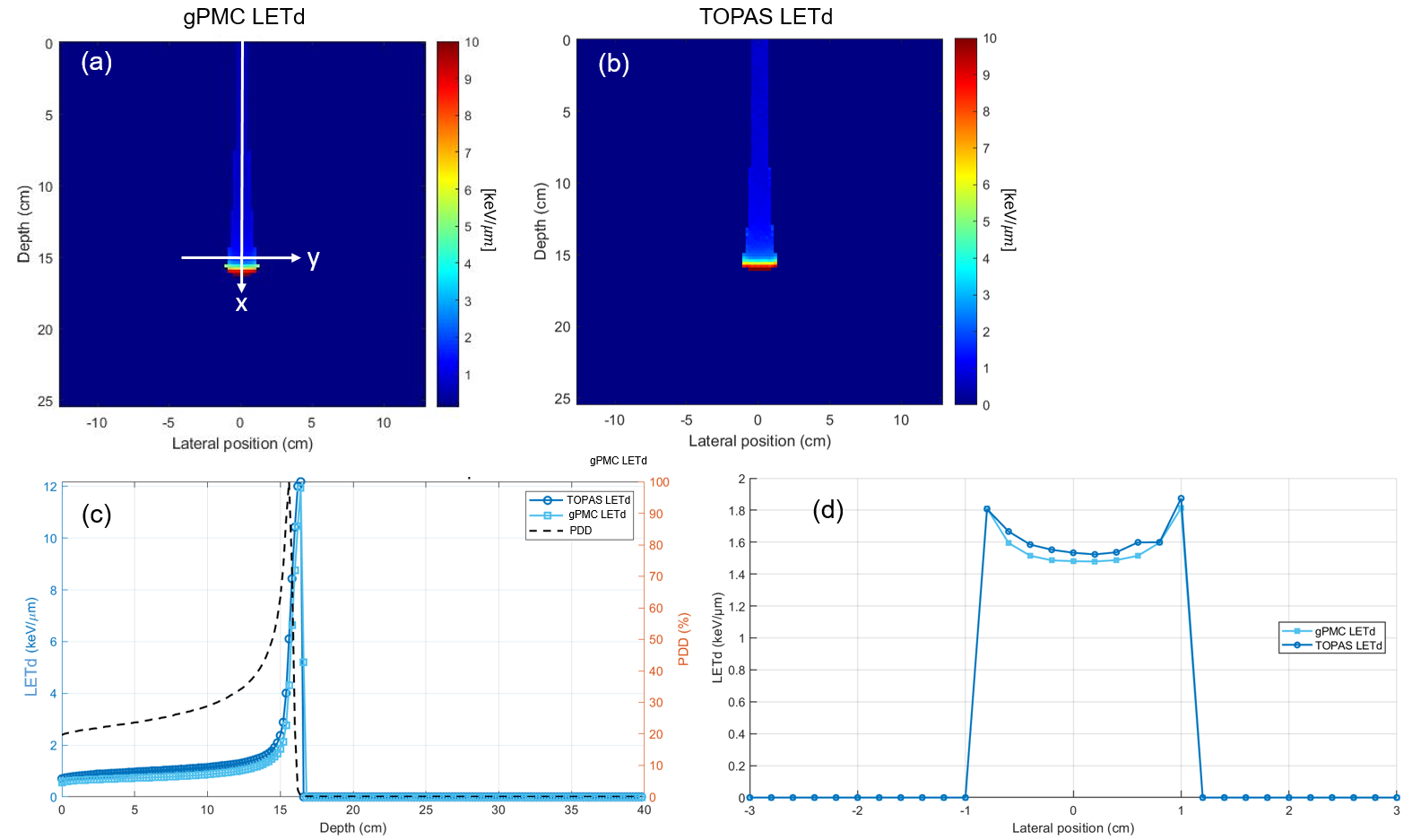


**Figure S1.** Validation of LET_d_ Monte Carlo (MC) simulations between gPMC and TOPAS. (a) 2D LET_d_ distribution simulated using gPMC. (b) 2D LET_d_ distribution simulated using TOPAS. (c) Comparison of depth–dose curves along the central axis (x-direction indicated in (a)), including the percentage depth dose (PDD) profile. (d) Comparison of lateral LET_d_ profiles at a depth of 15 cm (y-direction indicated in (a)).

1. Delivery efficiency estimation

We adopted a simple delivery time estimation model to estimate the delivery efficiency in Eq. (S1-S5) referring to typical parameters from the IBA Proteus®ONE system and realistic assumptions.

The total delivery time T is the sum of SST, SSWT, ELST and GRT for each control point $i$ within an arc with the resolution of 5.07 degree. For SST estimation, we considered the proton beam current of 1 nA, and minimum spot weighting of 0.01 MU, where the MU was defined by a cube with 10 to 20 cm depth of 1 L of water receiving a dose of 1 Gy when irradiated with 1000 MU (Ding *et al* 2016), and the spot scanning time is 5 ms/MU (Pfeiler *et al* 2018, Wuyckens *et al* 2024). We assumed SSWT along x-coordinate and y-coordinate were similar and can be approximated to be 2 ms per spot (Engwall *et al* 2022). The ELST is combined by total upward ELST and downward ELST, with the single operation of switching up takes 5.5 s, switching down takes 0.6 s (Li *et al* 2019). The GRT is used to estimate the rotation time between adjacent control points, $t$ is the ELST from the last energy layer in $\left( i-1 \right)$-th control point to the first energy layer in $i$-th control point, $GRT_{min}$ is the minimize rotation time between the adjacent control points determined by the beam angle resolution and the gantry rotation speed. Here we adopted a constant gantry rotation speed of 2 degree/s (Li *et al* 2019). For a gantry angular resolution of 5.07 degree, the minimum rotation time between two adjacent control points is $GRT_{min}=2.535 s$.

For Full-Arc and IMPT plans, multiple energy layers are delivered at each control point. A delivery-friendly sequencing strategy is adopted, where energy layers are delivered in a descending order within each control point, and energy increases only occur when transitioning between adjacent control points. Under this strategy, the upward energy switching time within a control point is avoided (i.e., $ELST_{upward}=0 s$ in Eq. (S4)), and the gantry rotation time between control points is $GRT_{i}=5.5 s$.

In contrast, MRPAT employs only one energy layer per control point, eliminating intra-control-point energy switching ($ELST_{i}=0 s$). As a result, the effective gantry rotation time between adjacent control points depends on the direction of energy change. If the energy increases between adjacent control points (upward switching), $GRT_{i}=5.5 s$. If the energy decreases (downward switching), $GRT_{i}=2.535 s$.

$T=\sum_{i=1}^{N} SST_{i}+SSWT_{i}+ELST_{i}+GRT_{i}$ (S1)

$SST_{i}=spot MU_{i} \times spot scanning time$ (S2)

$SSWT_{i}=spot number_{i} \times spot switching time$ (S3)

$ELST_{i}=ELST_{upward}+ELST_{downward}$ (S4)

$GRT_{i}=\left\{ \begin{aligned} t if t>GRT_{\min} \\ GRT_{min} if t\leq GRT_{\min} \end{aligned} \right.$ (S5)

**Reference**

Ding X, Li X, Zhang J M, Kabolizadeh P, Stevens C and Yan D 2016 Spot-Scanning Proton Arc (SPArc) Therapy: The First Robust and Delivery-Efficient Spot-Scanning Proton Arc Therapy *International Journal of Radiation Oncology*Biology*Physics* **96** 1107–16

Engwall E, Battinelli C, Wase V, Marthin O, Glimelius L, Bokrantz R, Andersson B and Fredriksson A 2022 Fast robust optimization of proton PBS arc therapy plans using early energy layer selection and spot assignment *Phys. Med. Biol.* **67** 065010

Granville D A and Sawakuchi G O 2015 Comparison of linear energy transfer scoring techniques in Monte Carlo simulations of proton beams *Phys. Med. Biol.* **60** N283–91

Li X, Liu G, Janssens G, De Wilde O, Bossier V, Lerot X, Pouppez A, Yan D, Stevens C, Kabolizadeh P and Ding X 2019 The first prototype of spot-scanning proton arc treatment delivery *Radiotherapy and Oncology* **137** 130–6

Pfeiler T, Bäumer C, Engwall E, Geismar D, Spaan B and Timmermann B 2018 Experimental validation of a 4D dose calculation routine for pencil beam scanning proton therapy *Zeitschrift für Medizinische Physik* **28** 121–33

Wuyckens S, Wase V, Marthin O, Sundström J, Janssens G, Borderias‐Villarroel E, Souris K, Sterpin E, Engwall E and Lee J A 2024 Efficient proton arc optimization and delivery through energy layer pre‐selection and post‐filtering *Medical Physics* **51** 4982–95
